# Thrombasthénie de Glanzmann: à propos de 11 cas

**DOI:** 10.11604/pamj.2015.21.268.6502

**Published:** 2015-08-11

**Authors:** Jean-Louis Ntumba Mukendi, Souad Benkirane, Azlarab Masrar

**Affiliations:** 1 Laboratoire d'Hématologie, Équipe de Recherche en Hématologie, Faculté de Médecine et de Pharmacie, Université Mohammed V, Rabat, Maroc; 2 Laboratoire Central d'Hématologie, Centre Hospitalier Ibn Sina, Rabat, Maroc

**Keywords:** Thrombasthénie de Glanzmann, plaquettes, hémorragie, Maroc, Glanzmann thrombasthenia, platelets, hemorrhage, Maroc

## Abstract

**Introduction:**

La thrombasthénie de Glanzmann est une pathologie hémorragique héréditaire rare due à une déficience ou un dysfonctionnement du complexe glycoprotéique IIb/IIIa de la membrane plaquettaire. Le but de notre étude est de décrire les caractéristiques démographiques, cliniques et biologiques d'une série de patients atteints de thrombasthénie de Glanzmann.

**Méthodes:**

C'est une étude portant sur tous les patients atteints de thrombasthénie de Glanzmann diagnostiqués au Laboratoire Central d'Hématologie du Centre Hospitalier Ibn Sina de Rabat au Maroc pendant la période allant du 1^er^ mars 2011 au 31 mars 2013, soit 25 mois. Après avoir recueilli les données épidémiologiques et cliniques de nos patients, nous avons réalisé une étude biologique comportant une numération plaquettaire et une agrégométrie.

**Résultats:**

11 patients ont présenté des profils agrégométriques compatibles à une TG. La majorité de ces malades étaient issus de mariages consanguins (54,5%) et originaires de régions situées dans le nord du Maroc. Le syndrome hémorragique s'est révélé principalement cutanéo-muqueux, avec une prédominance des gingivorragies (72,7%), des épistaxis (63,6%) et des ecchymoses (45,5%).

**Conclusion:**

Nos résultats ont montré que la thrombasthénie de Glanzmann est une pathologie relativement fréquente au Maroc.

## Introduction

La thrombasthénie de Glanzmann (TG) est un trouble hémorragique héréditaire dû à des anomalies quantitatives ou qualitatives des glycoprotéines GP IIb/IIIa de la membrane plaquettaire entraînant une agrégation plaquettaire anormale [1]. Cette pathologie a été décrite pour la première fois en 1918 par Glanzmann sous le nom de «thrombasthénie hémorragique héréditaire» [2]. Son expression clinique est souvent précoce, parfois dès la naissance, avec des hémorragies cutanéomuqueuses (purpura, épistaxis, gingivorragies, ménorragies, hémorragies digestives ou hématuries, …) [3]. Maladie rare, la TG est caractérisée par une hérédité autosomale récessive de distribution mondiale [4]. Toutefois, elle est relativement fréquente dans certaines régions du globe où les mariages consanguins sont communs [5]. Certaines régions du Maroc figurent parmi les foyers géographiques de la TG [4]. Notre travail a donc pour objectif de décrire les caractéristiques démographiques, cliniques et biologiques des patients atteints de TG diagnostiqués au Laboratoire Central d'Hématologie du Centre Hospitalier Ibn Sina (CHIS) de Rabat (Maroc).

## Méthodes

Le CHIS de Rabat est une structure constituée de 10 établissements hospitaliers de soins et d′hospitalisation. Son Laboratoire Central d'Hématologie traite les diverses demandes d'analyses dans les domaines de l'hématologie biologique provenant de ces différents hôpitaux. Le même laboratoire satisfait aussi des demandes externes. Dans ce contexte, notre étude transversale porte sur tous les patients atteints de TG diagnostiqués au Laboratoire Central d'Hématologie du CHIS de Rabat pendant la période allant du 1^er^ mars 2011 au 31 mars 2013, soit 25 mois.

### Interrogatoire

Pour chaque malade, nous avons recueilli, lors d'un interrogatoire préalable aux analyses et en complément des informations fournies par le praticien, les renseignements démographiques et cliniques, à savoir l’âge, le sexe, l'origine géographique, les antécédents hémorragiques personnels et/ou familiaux, le type de manifestations hémorragiques et d’éventuels notions de consanguinité et cas similaires vivants ou décédés. Par cas similaires, nous incluons les membres des familles présentant un syndrome hémorragique mais qui ne sont pas explorés.

### Exploration biologique

Les critères diagnostics appliqués dans cette étude sont une numération plaquettaire normale et un profil agrégométrique correspondant à une TG, soit une diminution voir une absence d'agrégation plaquettaire en réponse à l'ensemble des inducteurs à l'exception de la ristocétine.

### Etape pré-analytique

Les prélèvements ont été effectués, pour chacun des patients de notre étude, sur tubes anticoagulés: EDTA pour la numération sanguine; citrate de sodium (0,129 M) pour l’étude de la fonction plaquettaire.

### Etape analytique

#### Numération plaquettaire

La numération plaquettaire a été réalisée sur automate Sysmex XE 5000.

### Agrégométrie

L’étude de l'agrégation plaquettaire a été réalisée sur un thrombo-agrégomètre TA 4V (SD Medical, France) dans l'intervalle de 2 heures suivant le prélèvement. Ce test a été effectué sur plasma riche en plaquettes (PRP) avec 4 agonistes physiologiques: l'ADP, le collagène, l'acide arachidonique et la ristocétine.

#### Préparation du PRP

Nous avons procédé à une première centrifugation, lente (200 g pendant 15 minutes), afin d'obtenir le PRP. Une partie de celui-ci a subi, par la suite, une centrifugation rapide (3000 g pendant 15 minutes) visant l'obtention du plasma pauvre en plaquettes (PPP). Enfin, une numération plaquettaire a été effectuée sur le PRP. NB: l’étape de préparation du PRP s'est déroulée au moyen d'une centrifugation non réfrigérée à température de 22^°^C.

#### Utilisation de l'agrégomètre

Le thrombo-agrégomètre a été mis en marche 20 minutes avant la réalisation des tests fonctionnels plaquettaires pour qu'il atteigne une température de 37^°^C de façon stable. Ce, de sorte à reproduire les conditions physiologiques. Une fois que les 4 agonistes plaquettaires non encore utilisés et conservés à 4^°^C ont été décongelés extemporanément, nous avons ajusté leurs concentrations en fonction de celles voulues, au final, dans les cuvettes réactionnelles, à savoir 20 µM pour l'ADP, 2 µg/ml pour le collagène, 0,5 mg/ml pour l'acide arachidonique et 1,5 mg/ml pour la ristocétine. L'ensemble des inducteurs utilisés étaient de marque SD Medical, France. En dernier lieu, après avoir placé, au préalable, un agitateur magnétique tournant à 1000 trs/min dans chaque cuvette réactionnelle, nous avons d'abord établi le 100% d'agrégation sur le thrombo-agrégomètre avec le PPP et, ensuite, le 0% d'agrégation avec le PRP. Il ne restait plus qu’à injecter un agoniste plaquettaire par cuvette réactionnelle et laisser se développer le profil agrégométrique subséquent pendant 5 à 10 minutes. Le logiciel Thrombosoft 1.6 intégré dans notre automate a relevé la valeur de l'agrégation exprimée en pourcentage (%) à l'issue de 3 minutes ainsi que son maximum. La courbe obtenue a été validée en comparaison à un témoin sain non traité. L’étude statistique a été réalisée sur le logiciel Excel 2007.

## Résultats

**Figure 1:**
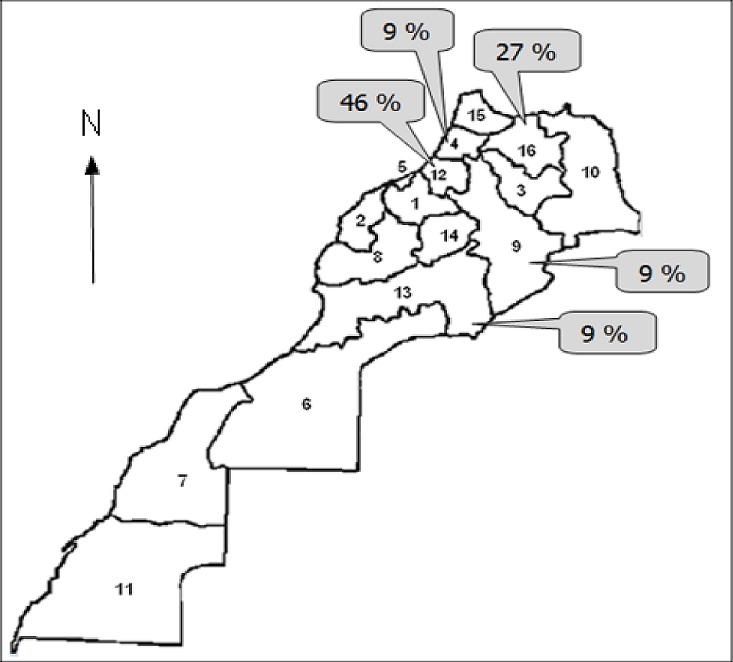
Répartition géographique de nos patients. Légende: régions administratives du Maroc: 1) Chaouia-Ouardigha; 2) Doukkala-Abda; 3) Fès-Boulemane; 4) Gharb-Chrarda-Beni Hssen; 5) Grand Casablanca; 6) Guelmim-Es Smara; 7) Laâyoune-Boujdour-Sakia el Hamra; 8) Marrakech-Tensift-El Haouz; 9) Meknès-Tafilalet; 10) Oriental; 11) Oued Ed-Dahab-Lagouira; 12) Rabat-Salé-Zemmour-Zaër; 13) Souss-Massa-Drâa; 14) Tadla-Azilal; 15) Tanger-Tétouan; 16) Taza-Al Hoceïma-Taounate

Nous avons diagnostiqué, au total, 11 patients atteints de TG. L’âge au moment du diagnostic variait de 1 à 15 ans, avec une médiane de 4 ans. A noter que 8 des patients ont été diagnostiqué à un âge inférieur ou égal à cette médiane. 6 patients étaient de sexe masculin et 5, de sexe féminin (sex-ratio = 1,2) (Tableau 1). Pour ce qui est de la répartition géographique, quatre familles provenaient de la région de Rabat-Salé-Zemmour-Zaër (avec 5 patients) et deux familles, de la région de Taza-Al Hoceïma-Taounate (avec 3 patients). Les trois familles restantes étaient originaires respectivement des régions de Gharb-Chrarda-Beni Hssen, Meknès-Tafilalet et Souss-Massa-Drâa (avec, pour chacune des familles, 1 patient, soit un total de 3 patients) (Figure 1). Parmi les 11 patients de notre série, 8 (72,7%) ont relaté des antécédents hémorragiques personnels et 5 (45,5%), des antécédents hémorragiques familiaux. De cette même population, 6 patients (54,5%) sont issus d'un mariage consanguin. Lors de l'enquête familiale, 2 cas similaires vivants ont été révélés, 1 parmi les ascendants et 1 dans les fratries. 4 cas similaires décédés ont également été identifiés, tous faisant partie des fratries.

**Tableau 1:** Répartition par sexe et âge de nos patients

Caractéristiques démographiques		Nombre	Pourcentage
**Sexe**	**Masculin**	6	54,5%
	**Féminin**	5	45,5%
**Age (ans)**	1-4	8	72,7%
	5-15	3	27,3%

**Figure 2:**
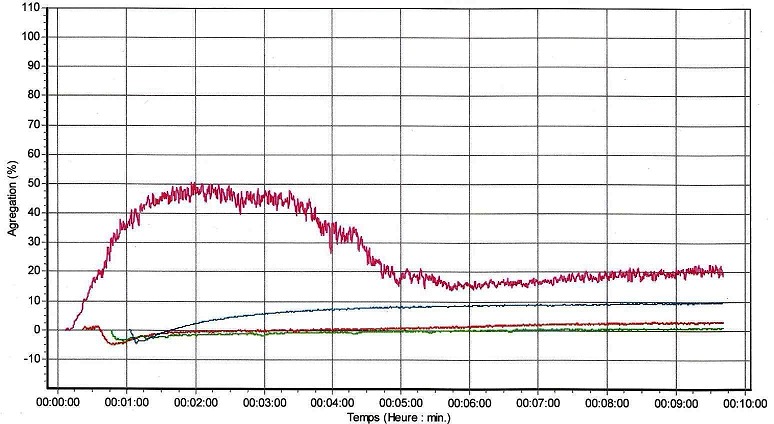
Profil agrégométrique du patient 1

**Figure 3:**
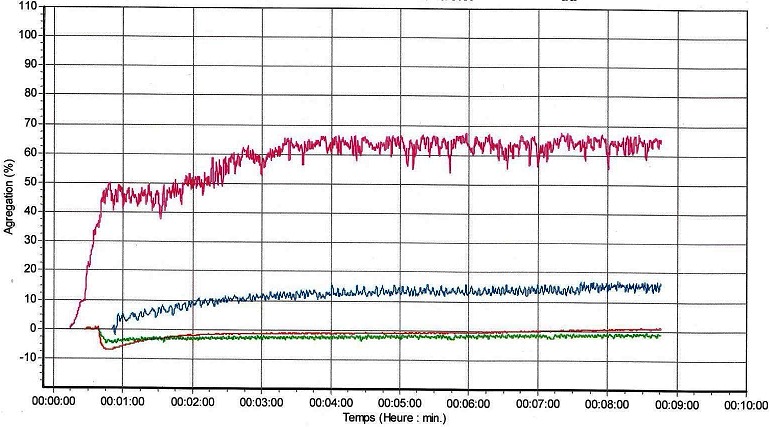
Profil agrégométrique du patient 2

**Figure 4:**
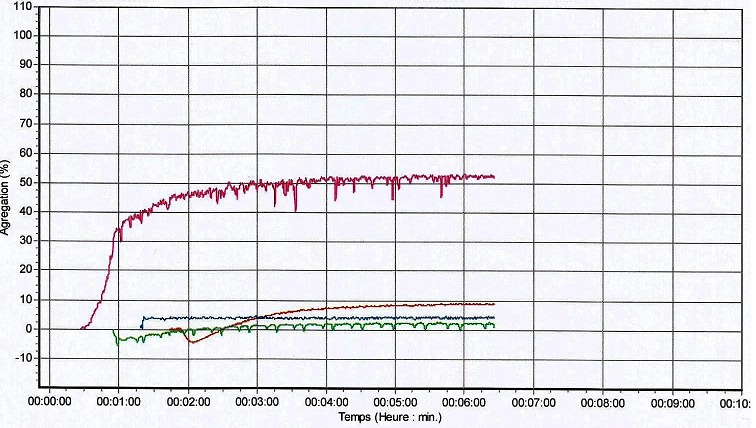
Profil agrégométrique du patient 3

**Figure 5:**
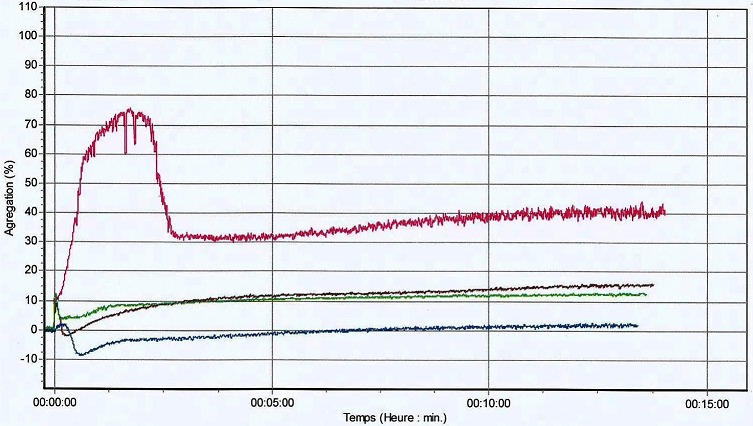
Profil agrégométrique du patient 4

Les gingivorragies constituent les signes cliniques les plus fréquents de notre série car rapportées par 8 patients (72,7%). Viennent ensuite les épistaxis dont ont souffert 7 patients (63,6%). A l'opposé, des cas d'hématome et de saignements du cordon ombilical, au point de vaccination, après circoncision, n'ont été rapportés, pour chacun de ces signes cliniques, que par un seul patient (9,1%) (Tableau 2). Les taux de plaquettes et les pourcentages d'agrégations plaquettaires des patients diagnostiqués sont consignés dans le Tableau 3. Quant aux profils agrégométriques de ces patients, 4 sont représentés sur les Figure 2, Figure 3, Figure 4, Figure 5. Sur celles-ci, les tracés de couleurs rose, rouge, verte et bleue correspondent respectivement à la ristocétine, au collagène, à l'ADP et à l'acide arachidonique excepté la figure 5 sur laquelle le tracé du collagène est bleu et celui de l'acide arachidonique, marron. Il faut noter que l'agrégation plaquettaire induite par la ristocétine s'est révélée réversible sur les profils agrégométriques de 6 patients dont ceux apparaissant sur les Figure 2, Figure 5. Par contre, sur les profils agrégométriques des 5 patients restants, les tracés dus au même agoniste ont montré une irréversibilité de l'agrégation, comme, entre autres, sur les Figure 3, Figure 4.

**Tableau 2:** Nombre et pourcentage de nos patients ayant manifesté au moins une fois des syndromes hémorragiques

Signes cliniques	Nombre	Pourcentage
Gingivorragie	8	72,7%
Epistaxis	7	63,6%
Ecchymose	5	45,5%
Pétéchies	3	27,3%
Hématurie	3	27,3%
Saignement du cordon ombilical	1	9,1%
Saignement au point de vaccination	1	9,1%
Saignement après circoncision	1	9,1%
Hématome	1	9,1%

**Tableau 3:** Taux de plaquettes et pourcentage d'agrégation de nos patients

Numération plaquettaire (G/L)		343 ± 112	
Pourcentage d'agrégation (%)		à t = 3 min	Max
**Agonistes**	**Ristocétine**	43,60 ± 19,62	57,65 ± 18,66
	**Collagène**	1,96 ± 3,74	9,52 ± 7,22
	**ADP**	0,70 ± 3,86	3,15 ± 3,83
	**Acide arachidonique**	4,15 ± 4,52	10,95 ± 5,67

## Discussion

En l'espace d'un peu plus de deux ans de prospections, nous avons diagnostiqué 11 cas de TG au Laboratoire Central d'Hématologie du CHIS de Rabat. Toujours au Maroc, le CHU Ibn Roch de Casablanca a enregistré le même nombre de cas de TG entre 1996 et 2011 (résultats non publiés). Et, en Tunisie, Ben Arabia a rapporté une série de 17 cas de TG traités au CHU Hédi Chaker de Sfax entre 1982 et 1999 [4]. Selon certaines estimations consensuelles, moins de 1000 cas de TG ont été diagnostiqués dans le monde [6]. Toutes ces statistiques reflètent la rareté de cette pathologie. Néanmoins, au vu de l'intervalle de temps court de nos investigations, le nombre de cas de TG que nous avons rencontré est important. A ce propos, nous pensons avoir bénéficié de l’évolution des méthodes de diagnostic par rapport aux études précédentes. La TG est couramment observée dans certaines ethnies au sein desquelles la consanguinité est courante à l'instar des Hindous du sud de l'Inde, des Juifs d'origine irakienne, des Gitans en France et des tribus nomades en Jordanie [2]. De même, la majorité des patients diagnostiqués dans le cadre de notre étude sont issus de mariages consanguins. Il sied de signaler que ce type de mariages fait partie intégrante de la culture marocaine, la recherche à l’échelle nationale publiée par Bouazzaoui en 1994 ayant abouti à un taux de consanguinité de 19,90%. Celui-ci est monté à 59,09% lors d'une autre étude nationale portant exclusivement sur des familles avec maladies autosomales récessives publiée par Jaouad en 2009 [7]. Par ailleurs, plus de deux tiers des patients de notre série étaient âgés de 4 ans ou moins au moment du diagnostic, comme rapporté précédemment [5]. Cela est certainement en lien avec la période d'expression clinique de la TG, laquelle correspond souvent à l'enfance [3].

Quant à sa répartition géographique, notre population d’étude provenait de 5 régions marocaines. Dans notre série, seul un cas originaire du sud du Royaume (région du Souss-Massa-Drâa) a été rencontré tandis que la maladie semble beaucoup plus fréquente dans le nord du pays et particulièrement dans la région de Rabat-Salé-Zemmour-Zaër (5 cas). Cette prédominance peut être due à la forte médicalisation du nord du Maroc : pour preuve, 4 des 5 CHU du Royaume y sont implantés. En plus, cette partie du pays comprend plusieurs zones à forte concentration urbaine, ce qui constitue un facteur favorisant. Toutefois, un biais de sélection ne peut être exclu, le CHIS de Rabat accueillant majoritairement les patients résidant dans le nord du Maroc. Sur le plan clinique, les gingivorragies constituaient le signe le plus courant dans notre série, suivies des épistaxis et des ecchymoses. Une tendance qui pourrait avoir été favorisée par une hygiène dentaire insuffisante [6]. Plus globalement, ces trois signes cliniques sont les plus fréquemment rapportés dans nombre de séries disponibles dans la littérature [8–10]. A l'opposé, certains signes cliniques à l'instar d'hématomes et de saignements après circoncision n'avaient été recensés qu’à une seule reprise dans notre étude. Les mêmes signes cliniques se sont avérés rares au cours de diverses études [8, 9]. En cas de TG, les hématomes profonds post-traumatiques sont exceptionnellement rapportés tandis que les hémorragies survenant post-circoncision ou post-extraction dentaire sont fréquentes [3]. Bien que les ménorragies fassent partie du tableau clinique classique de la TG [2, 5], nous n'en avons enregistrées aucune dans notre étude. Cela peut aisément se comprendre au vu de l’âge médian de notre population d’étude, bien en dessous de la puberté, période à partir de laquelle ce genre de problèmes survient. En outre, l'influence du contexte culturel et des valeurs morales peut pousser certaines patientes à taire des problèmes de règles trop abondantes [11]. Somme toute, le profil clinique de nos patients correspond aux descriptions de la littérature [3, 12].

Biologiquement, la TG se traduit par un taux de plaquettes normal avec un temps de saignement allongé. A noter que l’évaluation de ce dernier paramètre n'est plus recommandée, cette technique étant notamment opérateur-dépendante et invasive [3, 13]. Nous ne l'avons donc pas réalisée au cours de notre étude. Par contre, nous avons utilisé, comme test spécialisé, l'agrégométrie par variation de la transmission lumineuse. C'est la technique de référence d’évaluation de la fonction plaquettaire [13, 14]. Au travers de ce test, le diagnostic de la TG est suspecté en cas d'absence d'agrégation quel que soit l'agoniste utilisé et seule l'agglutination en présence de ristocétine est possible avec une première vague uniquement et une réversibilité constante [14], comme constaté sur les profils agrégométriques de nos patients. En règle générale, l'approche adoptée dans le cadre du diagnostic de confirmation de la TG en laboratoire diffère selon que le patient présente un trouble non spécifié de l'hémostase primaire ou que celui-ci est issu d'une famille affectée par cette maladie. Dans le premier cas, une série d'investigations pouvant comprendre, entre autres, l’étude du facteur Von Willebrand et la mesure du temps d'occlusion plaquettaire sera entreprise; quant à la seconde éventualité, elle justifie une approche plus directe, essentiellement constituée de l’étude des fonctions plaquettaires et de l'exploration des glycoprotéines plaquettaires par cytométrie en flux pour confirmer les résultats du test précédent. Toutefois, il est tout à fait possible d'opter pour une approche personnalisée [12]. C'est le cas dans notre travail en raison particulièrement de la non-disponibilité d'autres tests spécialisés. Cette limite nous a notamment empêchés de déterminer les formes de TG incluses dans notre série.

## Conclusion

Notre étude a montré que la TG est relativement fréquente au Maroc. Le diagnostic de cette maladie, qui nécessite une étroite collaboration entre le clinicien et le biologiste, y est confronté au principal obstacle de la non-disponibilité de l'ensemble des technologies requises. Dans ce contexte, il est avantageux d'investir dans la prévention en offrant notamment un conseil génétique relatif à cette maladie et, plus globalement, aux thrombopathies constitutionnelles.
